# Patient Safety during Propofol Sedation before and after Implementation of Capnography Monitoring

**DOI:** 10.3390/jcm12185959

**Published:** 2023-09-14

**Authors:** Volkan Baytaş, Çağıl Vural, Menekşe Özçelik, Rafael Torrejon Torres, Rhodri Saunders, Neslihan Alkış

**Affiliations:** 1 Department of Anaesthesiology and ICM, Faculty of Medicine, Ankara University, Ankara 06100, Türkiye; volkanbaytas@yahoo.com (V.B.);; 2 Department of Oral & Maxillofacial Surgery, Anaesthesiology Division, Faculty of Dentistry, Ankara University, Ankara 06100, Türkiye; 3 Health Economics, Coreva Scientific, 53639 Königswinter, Germany

**Keywords:** endoscopy, gastroenterology, quality of care, sedation, deep

## Abstract

Endoscopic procedures are routinely applied to cancer screening programs and surveillance. The preferred technique is usually deep sedation with propofol being a convenient agent allowing for a quicker patient recovery while maintaining a similar safety profile compared to traditional agents. However, adverse events, including respiratory depression and consequent undesirable cardiovascular side effects, may occur. The goal of this work is to evaluate the patient safety impact of adding capnography during endoscopic procedures under deep propofol sedation. Data were retrospectively collected from patients undergoing deep, procedural sedation for gastrointestinal (GI) endoscopy in October 2019 to January 2021 in a single Turkish university hospital. Included in the analysis were all adult patients classified by the American Society of Anesthesiologists (ASA) as I–IV, who were scheduled for GI endoscopy utilizing propofol alone or in combination. Data on 1840 patients were collected, of whom 1610 (730 pre- and 880 post-capnography implemention) met inclusion criteria. The primary outcome was a change in the composite incidence of mild oxygen desaturation (SpO_2_ 75–90% for <60 s), severe oxygen desaturation (SpO_2_ < 75% anytime or <90% for >60 s), bradycardia (<60 ppm), and tachycardia (>25% from baseline). Without capnography, on average, 7.5 events of the primary endpoint were observed per 100 procedures and 2.9 with additional capnography monitoring (*p* < 0.001). A significant reduction was observed for mild oxygen desaturation, with a resulting odds ratio of 0.25 (95% CI 0.14 to 0.46). ASA I patients had the highest difference in combined incidence of any oxygen desaturation of 5.85% in the pre-capnography group and 0.64% in the post-capnography group. Although procedural sedation using propofol is not associated with severe adverse events, the incidence of composite adverse events could be reduced with the addition of capnography monitoring.

## 1. Introduction

Endoscopic procedures are a common part of modern health care provisioning and prevention of death related with gastrointestinal (GI) system cancers. The most prominent use of endoscopy is the utilization for gastric and colorectal cancer (CRC) screening, with both forms of cancer being associated with a high death toll [1]. A survey from 2017 among Turkish primary care providers found that 90% of participants think that CRC is preventable with 78.9% considering screening as effective. The detection of gastric cancer during endoscopy is considered important [2]. For gastric cancer, though, screening was not considered feasible, with a 2011 study retrospectively analyzing gastroscopy patient data from a large Turkish teaching hospital showing that only between 3.91% and 6.26% of patients were diagnosed with early gastric cancer [2]. A survey by Tierney et al. investigated the patients who needed to undergo endoscopic procedures, finding that skills, personal manner, and adequacy of control of discomfort were the top ranking patient priorities [3].

To facilitate endoscopic procedures and increase patient comfort, the patients are generally placed under procedural sedation [4]. In a 2022 survey, 88.7% of Turkish gastroenterologists identified desaturation as a complication commonly associated with anaesthesia-related methods. Of those reporting causes of mortality (25/144 respondents), 32% reported respiratory arrest and 25% cardiac-related causes [5]. To ensure the safety of the patients, a number of guidelines has been developed, recommending training, anesthesiologist assistance, and an adequate target sedation level [4,5,6,7,8,9]. While procedural sedation is generally considered as safe, it may be associated with adverse events leading into respiratory compromise and also subsequent cardiovascular events [4,5,6,7,8,9]. These events are partially considered preventable, leading to standardized monitoring and staffing requirements as well as training recommendations [4,6,7,8,9].

An important factor for procedural sedation is the chosen sedative, which should be informed by evidence as well as the target sedation level. Compared to the traditional agents of midazolam and diazepam, propofol was found to be associated with a shorter procedure duration and an earlier discharge time [10,11], as well as a higher physician satisfaction [12]. In regard to adverse events, studies reported inconclusive results across studies for propofol and midazolam [12]. Propofol has been associated with a risk for cardiopulmonary events with 26 of 571 in 5 pooled studies and 38 of 646 in 7 pooled studies experiencing a desaturation or a bradycardia event, respectively [12].

To prevent cardiopulmonary events, non-invasive procedures, such as blood pressure (NIBP), electrocardiogram (ECG), pulse oximetry, and recently, capnography, are recommended to be utilized for patient surveillance [4,6,7,8,9]. While NIBP and ECG measure with a minimal overlap, capnography is directed at the prevention of similar adverse events than pulse oximetry. Where both methods have been compared, capnography was associated with a decreased risk in respiratory compromise [13]. In Turkey, though, a survey by Inal et al. published in 2022 reported none of the participating gastroenterologists use capnography [5].

Given the inconclusive evidence of propofol safety and a lack of solid evidence on the utilization of capnography in the Turkish setting this investigation was aimed to quantify the safety and impact of additional capnography monitoring during endoscopic procedures under deep sedation.

## 2. Materials and Methods

### 2.1. Design of the Service Evaluation

Data were collected from all therapeutic and diagnostic endoscopy procedures. The evaluation took place between October 2019 and January 2021 in a large Turkish university hospital. Inclusion criteria were all adults scheduled for an endoscopy procedure with procedural sedation utilizing propofol and who had a risk classification of American Society of Anesthesiologists (ASA I–IV). This evaluation was designed as pre-capnography and post-capnography implementation. We used portable respiratory monitor (Capnostream™ 35, Medtronic Ltd., Minneapolis, MN, USA). In our unit, anesthesiologists and anesthesia technicians monitored the capnography during the procedure. We used the Richmond Agitation Sedation Scale, and we applied propofol dose titration by targeting the −3, −4 level in the RASS score. No changes were made in the pre- and post-capnography implementation periods in terms of equipment used, unit staff, etc.

### 2.2. Data Collection

The data collected were selected after the literature review and expert opinion, focusing on respiratory compromise, cardiac events, and severe outcomes, as well as risk factors for the occurrence of such events. The identified parameters were programmed into an offline, Microsoft Excel™-based data collection tool, which was deployed on site. The data were first collected on paper forms and then entered into the tool by a Turkey-based independent third party (EBM research services). No patient identifiers were collected, and all data entered were secured using password protection.

This study was approved by the Ethics Committee of Ankara University (I5-336-21/1 June 2021/Yazihan, Prof. Dr.).

### 2.3. Collected Parameters

Collected parameters were split between three different sections: periprocedural data, adverse events, and interventions. Selected options for periprocedural data were procedure duration, date, procedure type (Table 1), sedative (propofol alone or in combination with other agents), the patient’s ASA risk classification (ASA I–IV), and age group (≤50, 51–60, 61–70, 71–80, >81).

The included adverse events were oxygen desaturation (SpO_2_ 75–90%) for <60 s, severe oxygen desaturation (SpO_2_ < 75% at any time or <90% for >60 s), prolonged apnea (>60 s), airway obstruction, bradycardia (<60 bpm), tachycardia (>25% from baseline), cardiovascular shock/collapse, cardiac arrest/absent pulse, and other, together with patient death. Included interventions were chest compressions, tracheal intubation, oral/nasal airway, bag-valve mask/assisted ventilation, laryngeal mask airway, continuous positive airway pressure (CPAP), use of reversal agents, and other. The events, interventions, and event definitions were taken from Mason et al. [14].

While multiple different adverse events and interventions were selectable, each event could only be selected once. Therefore, no repeat events could be collected, and a selected event may represent a single event or multiple events.

### 2.4. Additional Data

Collected on site, but not in the app, were additional risk factor data on age, gender, and body mass index (BMI), as well as outcomes not covered by the app: time until meaningful patient response to verbal stımuli and time until patient standing/mobile. On site and in the app, data were collected on the occurrence of an adverse event and the use of interventions. As no patient identifiers were stored, it was not possible to cross-reference onsite and app data one-to-one. These data are, therefore, assessed separately.

### 2.5. Primary Outcome

To conclude any clinical significance of the findings, the potential adverse events were combined as a primary outcome. The events were defined after the literature review and expert opinion, identifying those events that are preventable using both pulse oximetry and capnography. This composite outcome included: oxygen desaturation (SpO_2_ 75–90% for <60 s), severe oxygen desaturation (SpO_2_ > 75% anytime or <90% for >60 s), bradycardia (<60 bpm), and tachycardia (>25% from baseline). A 20% reduction in the incidence of the primary outcome was set as the threshold for a meaningful difference in the event occurrence. The patient population required was calculated based on a 20% alpha and 5% beta error, leading to an estimated patient count of 666 for each pre- and post-capnography implementation group.

### 2.6. Data Analysis

Upon completion of each part, pre- and post-capnography implementation, a copy of the data collection tool was created and uploaded to a secure cloud provider for download and analysis. The data were analyzed for the incidence of events, the odds ratio for event occurrence between pre- and post-capnography implementation, and the occurrence by a periprocedural risk factor.

The impact of potential confounders on the primary outcome was assessed via a multivariable logistic regression model, including age (8 ordinal categories), BMI (numerical continuous), ASA level (4 ordinal categories), gender (binary), adverse event occurrence (binary), and capnography (binary) to predict the duration. Linear regression analysis was performed in RStudio [15] (R version 4.1.1) utilizing the stats package (4.1.1).

## 3. Results

### 3.1. Population Statistics

**Figure 1:**
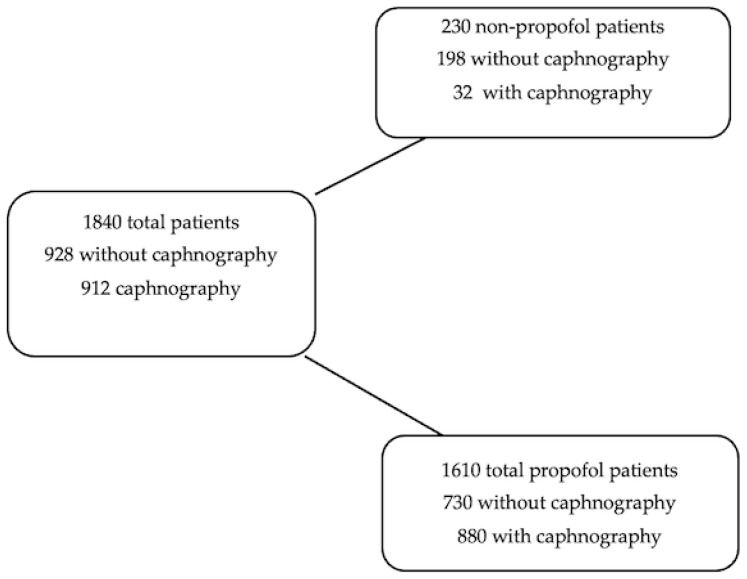
Flowchart of patients included in the study.

Data on 1840 patients were collected, of which 928 were without and 912 with capnography. Excluding sedative agents other than propofol, data on 730 procedures pre- and 880 procedures post-capnography were considered for analysis, surpassing the target of 666 per the evaluation stage (Figure 1). Of those procedures registered in the data collection tool, the majority were gastroscopies and colonoscopies, either alone or in combination, followed by endoscopic retrograde cholangiopancreatography (ERCP) and endoscopic diagnostic ultrasonography (Table 2). In the baseline, 4.93% of the procedures were also esophagoscopies with endoscopic diagnostic ultrasonography, which were not recorded during the capnography part of the data collection. Otherwise, the number of gastroscopies was substantially higher in the capnography group.

For patients between 61 and 70 years, 2.51% more patients were in the capnography group, while this difference remained under one percent for the other age groups. In regard to the ASA classifications, there was a moderate difference between patient populations; while the distribution of ASA I and II patients is nearly equal in the non-capnography population, the amount of ASA II patients is 1.66-fold the number of ASA I patients in the capnography population.

Only patients receiving propofol were considered for this evaluation. Of the 1840 initially entered, 230 were excluded due to the usage of sedatives other than propofol, resulting in 1610 propofol patients included for the analysis.

### 3.2. Adverse Events

In total, 55 adverse events relevant to the primary outcome were recorded during the 730 non-capnography procedures and 26 after in the 880 procedures with capnography, corresponding with 7.5 and 2.9 events per 100 procedures, respectively (Table 3). The most observed events were mild oxygen desaturations, which were occurring in 6.03% of procedures in non-capnography and 1.59% of procedures with capnography. Events other than the primary outcome were rarely observed, with two prolonged apnea and two airway obstructions (0.27% of procedures each) being recorded in the baseline procedures, and one prolonged apnea (0.11% of procedures) in the capnography procedures. Considering all recorded adverse events, a significant (*p* < 0.001) relative reduction of 60.79% was observed in the capnography group.

Considering patient risk groups, except for bradycardia and the severe oxygen desaturations observed in ASA II patients, all incidences were reduced. The highest difference was observed in ASA I patients, where the combined incidence of the primary outcome was 5.85% during the baseline and 0.64% after the addition of capnography. Given that the patient count is not sufficient to conclude significance, no *p*-value is supplied.

Except for bradycardia, the events comprising the primary outcome were reduced after the implementation of capnography. For moderate oxygen desaturations, this difference was even estimated to be significant, with an associated odds ratio of 0.25 (95% CI 0.14 to 0.46) (Table 4). The other three events, which were considered a primary outcome, did not show significance.

### 3.3. Procedure Duration

The procedures took on average 20.14 min in the capnography group and 21.87 min in the non-capnography group (Table 5). Comparing the duration of procedures with and without a primary outcome of adverse events, procedures without adverse events were shorter on average for both males (2.65 min shorter) and females (7.39 min shorter). For the overall population, this averaged to 4.95 and 4.96 min in the pre- and post-capnography implementation group, respectively, corresponding to a 23–25% increase in the procedure durations. This indicates a negative impact of an adverse event occurrence on the procedure length.

**Figure 2:**
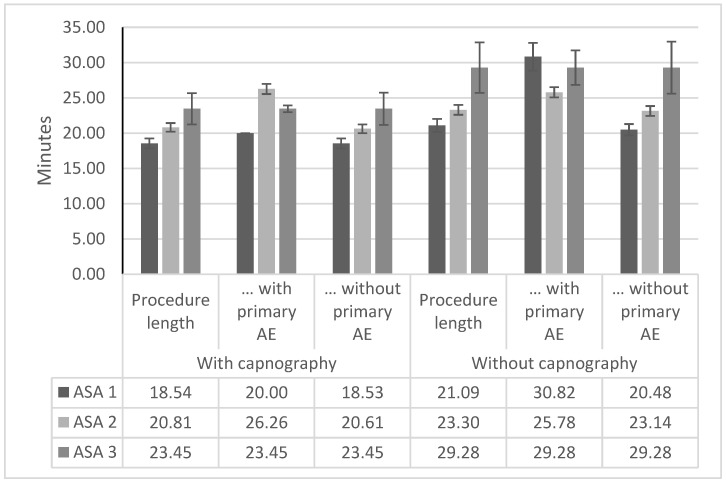
Procedure duration by risk classification and event occurrence. Procedure duration in minutes by risk classification, capnography group, and risk classification. In both groups and for all ASA classifications, the patients experiencing adverse events were on average associated with an increased procedure duration. No error bar supplied for ASA 1 capnography patients with adverse events as only one event occurred in the population. AE, adverse event. ASA, American Society of Anesthesiologist.

Considering the procedure duration by risk classification and event occurrence, the highest difference between the non-capnography and the capnography group is evident for patients classified as ASA 1 (Figure 2). Here, while only one patient receiving additional capnography monitoring experienced an adverse event during the procedure, the additional time required in the non-capnography group was substantial. In the non-capnography group, the difference in the average procedure duration between ASA 1 patients having an uncomplicated procedure and patients with an adverse event as described in the primary outcome was more than ten minutes, which translates to an increase of over 50% in the average procedure time.

After the procedure, the time until a meaningful response was 4.34 and 5.38 min in the capnography and non-capnography group, respectively. For time to standing, however, the trend was reversed, with the average time to standing in the non-capnography group being almost 15 min less. A potential confounding parameter could be the change in protocol due to the onset of the SARS-CoV-19 pandemic, which escalated only at the end of the non-capnography group data collection and affected the capnography group in full.

To adjust for the individual risk factors contributing to the procedure duration, a multivariable linear model was created from the used dataset (Table 6). The model created was associated with a high likelihood (*p* < 0.001) of a statistical relationship between risk factors and procedure duration. The model intercept was calculated at 21.70 min for a male patient younger than 20, who was classified as ASA 1, with a BMI of 26, no adverse event, and no capnography used. Of the parameters included in the model, it was estimated that capnography (a reduction in procedure length by 2.50 min), age >90 years (an increase in procedure length by 9.47 min), and the occurrence of an adverse event (an increase in procedure length by 3.49 min) are significant predictors (*p* ≤ 0.05) of a change in the procedure duration. For ASA, while not reaching significance, the procedure duration increased with higher classification. The BMI had only a low impact on the procedure length, with every point increase decreasing the procedure length by 3 min and vice versa for each point of decreased BMI.

### 3.4. Interventions

Overall, only two pre-defined interventions, one tracheal intubation and one utilization of reversal agents, each accounting for 0.13% of the baseline procedures, were recorded during the data collection. The vast majority of interventions were classified as other and utilized in 5.06% and 1.93% of non-capnography and capnography procedures, respectively. Other interventions include minor interventions, like oxygen augmentation, head repositioning, and tactile and verbal stimuli to the patient. The utilization of these interventions was in all cases linked to adverse events occurring during the procedure.

## 4. Discussion

The addition of the capnography monitor for procedures utilizing procedural sedation with propofol showed significantly increased patient safety in regard to the events as defined in the primary outcome. This was predominantly due to the significant reduction in the odds of a mild oxygen desaturation event (OR: 0.25, 95% CI 0.14 to 0.46). Compared to other Turkish cohorts, the number of events observed during the baseline is lower than in previous publications reporting on propofol sedation. Gurbulak et al. compared the safety of propofol with midazolam/medperidine sedation for colonoscopic procedures, reporting an 8.1% incidence of oxygen desaturation events in the propofol arm [16]. In our evaluation, if only colonoscopy is considered, only 4.03% of procedures were associated with any oxygen desaturation event (mild and severe desaturations). In the same study, bradycardias also were registered, but none were found in the propofol arm [16]. This matches our analysis, as only a single adverse event of bradycardia was observed for the 264 patients receiving gastroscopy only.

A similar study by Uzman et al. compared the same sedation regimen for upper gastrointestinal endoscopy [17]. Here, 7 out of 50 patients (14%) in the propofol cohort experienced oxygen desaturations [17]. In our evaluation, the observed incidence for any oxygen desaturation during upper gastrointestinal tract endoscopy was 15.31% in the ERCP procedure and 6.21% for gastroscopy, with the other procedures having a too low patient count to draw a conclusion. Both our evaluation and the work of Uzman et al. use an equivalent definition of oxygen desaturations. While the incidence of oxygen desaturations during ERCP procedures is comparable to the incidence observed by Uzman et al., the incidence observed during gastroscopy procedures is lower. This could be explained by a potentially higher-risk procedure mix in the population of Uzman et al., but which was not reported in detail by the authors [17].

In our finding, patients classified as ASA 1 benefited the most from capnography monitoring, which was surprising given that patients with this classification are generally considered to have an absence of notable risk factors for respiratory complications. The finding, though, is in line with the observations of Vargo et al. and was also observed in a similar evaluation published by Bisschops et al. [18,19]. This suggests that more emphasis should potentially be put on the monitoring of low-risk patients, as the average procedure duration with an adverse event was 1.5-fold as long as without any adverse event for ASA 1 patients (Figure 2). This might accrue a substantial resource burden given the 4.52% incidence in the non-capnography group compared to the 0.32% incidence in the capnography group (Table 3).

A large retrospective chart review of a single hospital by Kilic et al. also investigated the safety of sedation during ERCP but additionally considered endoscopic ultrasound while administering ketamine–propofol as a sedative agent [20]. The main focus of Kilic et al. was to determine the link between safety and obesity levels [20]. Similar to our work, Kilic et al. added capnography to the monitoring protocol for their patients but used a different definition of oxygen desaturations (<80% for 3 min), all of which would be classified as severe within our publication [20]. Considering only overweight patients (BMI 25–30), the fraction of 7.7% of patients experiencing an oxygen desaturation event is higher in our reported incidence for severe desaturations, which is 1.02% and 1.05% before and after implementation of capnography, respectively [20]. The incidence as measured in our population may be lower due to the more general population at a lower risk for oxygen desaturation, but this analysis is not possible due to the non-inclusion of weight. For ERCP alone, Koruk et al. analyzed the safety of propofol in conjunction with midazolam or dexmedetomidine [21]. While this study did not report on the occurrence of oxygen desaturations, it accounted for bradycardias (>20% baseline) and reported that none occurred [21]. Given the smaller population of 20 patients per arm in the population analyzed by Koruk et al. and the reported incidence of 2.04% in our work, it is possible that the results are comparable between both populations [21].

Considering the efficacy of capnography monitoring, the results of this work show similar tendencies as the meta-analysis by Saunders et al. [13]. For moderate oxygen desaturations, the incidence as found by our work is lower (OR: 0.25, 95% CI 0.14 to 0.46) than the value reported by the meta-analysis (OR: 0.77, 95% CI 0.67 to 0.89), while also being considered significant [13]. The risk for severe desaturations, however, is on a similar magnitude than the reported value from the meta-analysis (OR: 0.59, 95% CI 0.43 to 0.81) and the value not reaching significance (OR: 0.71, 95% CI 0.24 to 2.1) [13]. The risk for bradycardia was elevated with capnography but not reaching significance, similar to the meta-analysis [13].

Given the magnitude in the difference in event occurrence between the baseline and the capnography arm, aside from the patient safety benefit, the hospital resource burden could also be reduced. In a payer survey from 2018 for France, Germany, Italy, the UK, and the US, oxygen desaturations were associated with an average cost between USD 53 and USD 130 depending on the severity and country [22].

For the sake of patient satisfaction, a better control over the procedure may also increase the adherence to endoscopy screening protocols, given that skill and adequacy of discomfort were high ranking across patient expectations on endoscopy procedures [3]. In Turkey, a survey conducted by the World Health Organization (WHO) found that only 12.1% of respondents aged 50–70 had a colonoscopy during the previous 10 years [23].

## 5. Conclusions

During propofol sedation, the addition of a capnography monitor can significantly reduce the occurrence of the events of respiratory compromise as defined within the primary outcome of this investigation. After the implementation of capnography, both a reduction in adverse events as well as a reduction in interventions were recorded, showing the potential to reduce the burden of the procedure to the clinicians.

## 6. Limitations

This work was designed as a retrospective cohort evaluation and is, therefore, not comparable with a full study. The patients were not randomized, and healthcare staff were not blinded to the monitor. In addition, due to training on the usage of the capnography monitor, it cannot be excluded that awareness for respiratory compromise was increased. This work was also conducted during the onset of the SARS-CoV-2 pandemic; therefore, a potential bias due to patient selection and recording is possible, but this could not be foreseen and accounted for by the design.

Additionally, the high sample size of 1610 patients and the resulting large dataset shows promising results on the utilization of capnography during propofol-based sedation.

## 7. Disclosures

RTT is an employee of and RS is the owner of Coreva Scientific GmbH & Co KG, which received consultancy fees for performing, analyzing, and communicating the work presented here.

## Figures and Tables

**Table 1:** Procedure types.

Procedure Type
Percutaneous endoscopic gastrostomy
Endoscopic esophageal variceal ligation
Endoscopic cysto-gastrostomy
Endoscopic retrograde cholangiopancreatography
Percutaneous transhepatic cholangiography
Endoscopic submucosal dissection
Endoscopic mucosal dissection
Endoscopic diagnostic ultrasonography
Gastroscopy
Esophagoscopy
Esophagoscopy and endoscopic submucosal dissection
Esophagoscopy and endoscopic diagnostic ultrasonography
Colonoscopy
Colonoscopy and Gastroscopy

**Table 2:** Patient population.

Parameter	Non-Capnography N Patients	Capnography N Patients	*p*-Values
Total	730	880	
Procedure			<0.05
Gastroscopy	177 (24.25%)	311 (35.34%)	
Colonoscopy	124 (16.99%)	140 (15.91%)	
Gastroscopy + Colonoscopy	254 (34.79%)	288 (32.73%)	
PEG	2 (0.27%)	1 (0.11%)	
ERCP	98 (13.42%)	95 (10.80%)	
ESD	3 (0.41%)	0 (0.00%)	
EMD	3 (0.41%)	3 (0.34%)	
EDU	31 (4.25%)	41 (4.66%)	
Escopy + EUD	36 (4.93%)	1 (0.11%)	
Escopy + ESD	2 (0.27%)	0 (0.00%)	
Age group			0.902
≤50 years	273 (37.40%)	328 (37.27%)	
51–60 years	191 (26.16%)	224 (25.45%)	
61–70 years	165 (22.60%)	221 (25.11%)	
71–80 years	79 (10.82%)	88 (10.00%)	
>80 years	22 (3.01%)	19 (2.16%)	
ASA classification			0.388
ASA I	354 (48.49%)	315 (35.80%)	
ASA II	339 (46.44%)	526 (59.77%)	
ASA III	37 (5.07%)	38 (4.32%)	
ASA IV	0 (0.00%)	1 (0.11%)	
Sedative			<0.05
Propofol	660 (90.41%)	819 (93.07%)	
Propofol + fentanyl	28 (3.84%)	33 (3.75%)	
Propofol + ketamine	12 (1.64%)	5 (0.57%)	
Propofol + midazolam	18 (2.47%)	6 (0.68%)	
Propofol + fentanyl + midazolam	12 (1.64%)	17 (1.93%)	

ASA, American Society of Anesthesiologists; PEG, percutaneous endoscopic gastrostomy, ERCP, endoscopic retrograde cholangiopancreatography; ESD, endoscopic submucosal dissection; EMD, endoscopic mucosal dissection; EDU, endoscopic diagnostic ultrasonography; Escopy, esophagoscopy.

**Table 3:** Adverse events relevant to the primary outcome.

Parameter	Mild Oxygen Desaturation		Severe Oxygen Desaturation		Bradycardia		Tachycardia	
N Events (% of Procedures with Event)								
	Without Capnography	With Capnography	Without Capnography	With Capnography	Without Capnography	With Capnography	Without Capnography	With Capnography
Total	44 (6.03%)	14 (1.59%)	7 (0.96%)	6 (0.68%)	2 (0.27%)	5 (0.57%)	2 (0.27%)	1 (0.11%)
Procedure								
Gastroscopy	10 (5.65%)	3 (0.96%)	1 (0.56%)	1 (0.32%)	0	0	0	0
Colonoscopy	3 (2.42%)	1 (0.71%)	2 (1.61%)	0	0	1 (0.71%)	0	0
Gastroscopy + Colonoscopy	11 (4.33%)	5 (1.74%)	1 (0.39%)	2 (0.69%)	0	2 (0.69%)	0	0
PEG	0	0	0	0	0	0	0	0
ERCP	14 (14.29%)	4 (4.21%)	1 (1.02%)	1 (1.05%)	2 (2.04%)	1 (1.05%)	1 (1.02%)	1 (1.05%)
ESD	1 (33.33%)	0	0	0	0	0	0	0
EMD	1 (33.33%)	0	0	0	0	0	0	0
EDU	0	1 (2.44%)	0	2 (4.88%)	0	1 (2.44%)	0	0
Escopy + EDU	3 (8.33%)	0	1 (2.78%)	0	0	0	0	0
Escopy + ESD	1 (50.00%)	0	1 (50.00%)	0	0	0	1 (50.00%)	0
Risk classification								
ASA I	16 (4.52%)	1 (0.32%)	3 (0.85%)	0	1 (0.28%)	1 (0.32%)	0	0
ASA II	23 (6.78%)	10 (1.90%)	1 (0.29%)	6 (1.14%)	1 (0.29%)	4 (0.76%)	1 (0.29%)	1 (0.19%)
ASA III	5 (13.51%)	3 (7.89%)	3 (8.11%)	0	0	0	1 (2.70%)	0
ASA IV	0	0	0	0	0	0	0	0

ASA, American Society of Anesthesiologists; PEG, percutaneous endoscopic gastrostomy; ERCP, endoscopic retrograde cholangiopancreatography; ESD, endoscopic submucosal dissection; EMD, endoscopic mucosal dissection; EDU, endoscopic diagnostic ultrasonography; Escopy, esophagoscopy.

**Table 4:** Odds ratio for adverse events.

Adverse Event	Odds Ratio (95% CI)
Mild oxygen desaturation (75–90% for <60 s)	0.25 (95% CI 0.14 to 0.46)
Severe oxygen desaturation, <75% for any duration or <90% for over 60 s	0.71 (95% CI 0.24 to 2.12)
Bradycardia	2.08 (95% CI 0.40 to 10.75)
Tachycardia, >25% from baseline	0.41 (95% CI 0.04 to 4.58)

**Table 5:** Procedure and recovery duration by event occurrence.

	All *n* = 1609		Male *n* = 748		Female *n* = 861	
	With Capnography	Without Capnography	With Capnography	Without Capnography	With Capnography	Without Capnography
Events [N]	23	48	12	14	11	34
Procedure length (min)	20.14 ± 13.4	21.87 ± 27.23	20.85± 13.19	23.3 ± 13.81	19.43 ± 13.59	20.8 ± 33.88
… with primary outcome event (min)	24.96 ± 15.07	26.5 ± 22.39	23.42 ± 10.66	27.57 ± 14.6	26.64 ± 19.21	26.06 ± 25.09
… without primary outcome event (min)	20.01 ± 13.34	21.54 ± 27.53	20.77 ± 13.26	23.1 ± 13.76	19.25± 13.4	20.34 ± 34.54
Meaningful response (min)	4.34 ± 2.8	5.38 ± 3.93	4.42 ± 3.08	5.33 ± 3.91	4.26 ± 2.49	5.41± 3.95
… with primary outcome event (min)	6.91 ± 6.54	8.23 ± 4.47	8.5 ± 8.53	8.36 ± 4.53	5.18 ± 2.79	8.18± 4.51
… without primary outcome event (min)	4.27 ± 2.6	5.18± 3.81	4.3 ± 2.72	5.19 ± 3.83	4.23 ± 2.48	5.17 ± 3.8
Standing up (min)	57.48 ± 16.67	42.63 ± 14.15	57.16 ± 16.79	43.11 ± 14.5	57.81 ± 16.57	42.27 ± 13.89
… with primary outcome event (min)	70.83 ± 17.41	46.02 ± 13.68	69.92 ± 17.93	42.79 ± 10.6	71.82 ± 17.65	47.35 ± 14.7
… without primary outcome event (min)	57.12 ± 16.52	42.39 ± 14.16	56.8 ± 16.64	43.13 ± 14.67	57.45 ± 16.41	41.82 ± 13.74

Relationship between procedure and recovery duration and the occurrence of any adverse event as defined by the primary outcome. Mean with standard deviation in parentheses shown.

**Table 6:** Linear multivariate regression model.

Parameter	Estimate	Standard Error	*p*-Value
ASA1	21.70	3.19	<0.001
ASA2	0.45	0.79	0.57
ASA3	3.16	1.75	0.07
ASA4	8.08	13.89	0.56
Adverse event	3.49	1.70	<0.05
Capnography	−2.50	0.70	<0.001
Age < 30	−0.40	3.06	0.90
Age < 40	−2.90	2.90	0.32
Age < 50	0.34	2.84	0.90
Age < 60	1.66	2.82	0.56
Age < 70	4.35	2.84	0.13
Age < 80	3.27	2.91	0.26
Age < 90	9.47	3.42	<0.01
Female	−1.27	0.69	0.07
BMI(Median 26)	−0.03	0.07	0.66

Residual standard error: 13.72 on 1587 degrees of freedom (7 observations deleted due to missing data), Multiple R-squared: 0.05528, Adjusted R-squared: 0.04694, F-statistic: 6.632 on 14 and 1587 DF, *p*-value: 2.9 × 10^−13^.

## Data Availability

Not applicable.
